# Healthcare resource utilization and costs of outpatient follow-up after liver transplantation in a university hospital in São Paulo, Brazil: cost description study: Utilização de recursos em saúde e custos do acompanhamento ambulatorial pós-transplante de fígado em um hospital universitário em São Paulo, Brasil: estudo de descrição de custos

**DOI:** 10.1590/1516-3180.2013.7000011

**Published:** 2014-12-19

**Authors:** Patricia Coelho de Soárez, Amanda Nazareth Lara, Ana Marli Christovam Sartori, Edson Abdala, Luciana Bertocco de Paiva Haddad, Luiz Augusto Carneiro D’Albuquerque, Hillegonda Maria Dutilh Novaes

**Affiliations:** I DDS, MPH, PhD. Adjunct Professor, Department of Preventive Medicine, Faculdade de Medicina da Universidade de São Paulo (FMUSP), São Paulo, Brazil.; II MD. Attending Physician, Infectious and Parasitic Diseases Clinic, Hospital das Clínicas (HC), Faculdade de Medicina da Universidade de São Paulo (FMUSP), São Paulo, Brazil.; III MD, MSc, PhD. Attending Physician, Infectious and Parasitic Diseases Clinic, Hospital das Clínicas (HC) Faculdade de Medicina da Universidade de São Paulo (FMUSP), São Paulo, Brazil.; IV MD, MSc, PhD. Attending Physician, Digestive Organ Transplantation Service, Department of Gastroenterology, Faculdade de Medicina da Universidade de São Paulo (FMUSP), São Paulo, Brazil.; V MD, MSc. Attending Physician, Digestive Organ Transplantation Service, Department of Gastroenterology, Faculdade de Medicina da Universidade de São Paulo (FMUSP), São Paulo, Brazil.; VI MD, MSc, PhD. Titular Professor, Digestive Organ Transplantation Service, Department of Gastroenterology, Faculdade de Medicina da Universidade de São Paulo (FMUSP), São Paulo, Brazil.; VII MD, MSc, PhD. Associate Professor, Department of Preventive Medicine, Faculdade de Medicina da Universidade de São Paulo (FMUSP), São Paulo, Brazil.

**Keywords:** Health care costs, Costs and cost analysis, Liver transplantation, Liver failure, acute, Ambulatory care, Custos de cuidados de saúde, Custos e análise de custo, Transplante de fígado, Falência hepática aguda, Assistência ambulatorial

## Abstract

**CONTEXT AND OBJECTIVE:**

Data on the costs of outpatient follow-up after liver transplantation are scarce in Brazil. The purpose of the present study was to estimate the direct medical costs of the outpatient follow-up after liver transplantation, from the first outpatient visit after transplantation to five years after transplantation.

**DESIGN AND SETTING:**

Cost description study conducted in a university hospital in São Paulo, Brazil.

**METHODS:**

Cost data were available for 20 adults who underwent liver transplantation due to acute liver failure (ALF) from 2005 to 2009. The data were retrospectively retrieved from medical records and the hospital accounting information system from December 2010 to January 2011.

**RESULTS:**

Mean cost per patient/year was R$ 13,569 (US$ 5,824). The first year of follow-up was the most expensive (R$ 32,546 or US$ 13,968), and medication was the main driver of total costs, accounting for 85% of the total costs over the five-year period and 71.9% of the first-year total costs. In the second year after transplantation, the mean total costs were about half of the amount of the first-year costs (R$ 15,165 or US$ 6,509). Medication was the largest contributor to the costs followed by hospitalization, over the five-year period. In the fourth year, the costs of diagnostic tests exceeded the hospitalization costs.

**CONCLUSION:**

This analysis provides significant insight into the costs of outpatient follow-up after liver transplantation due to ALF and the participation of each cost component in the Brazilian setting.

## RESUMO

**CONTEXTO E OBJETIVO:**

Dados sobre os custos do seguimento ambulatorial pós-transplante de fígado são escassos no Brasil. O objetivo do presente estudo foi estimar os custos diretos médicos do seguimento ambulatorial pós-transplante de fígado a partir da primeira visita ambulatorial pós-transplante até cinco anos após o transplante.

**TIPO DE ESTUDO E LOCAL:**

Estudo de descrição de custos realizado em um hospital universitário em São Paulo, Brasil.

**MÉTODOS:**

Dados de custos estavam disponíveis para 20 adultos que foram submetidos a transplante de fígado devido a insuficiência hepática aguda (IHA) de 2005 a 2009. Os dados foram retrospectivamente obtidos em prontuários médicos e no sistema de informação contábil hospitalar de dezembro de 2010 a janeiro de 2011.

**RESULTADOS:**

A média de custo por paciente/ano foi de R$ 13.569 (US$ 5.824). O primeiro ano de acompanhamento foi o mais caro, R$ 32.546 (US$ 13,968), e medicação foi o principal impulsionador dos custos totais, respondendo por 85% dos custos totais no período de cinco anos e 71,9% dos custos totais do primeiro ano. No segundo ano pós-transplante, os custos médios totais foram cerca da metade do montante de custos do primeiro ano (R$ 15.165 ou US$ 6,509). Medicação foi o maior contribuinte para os custos seguido da internação, no período de cinco anos. No quarto ano, os custos dos testes diagnósticos superam os custos de internação.

**CONCLUSÃO:**

Esta análise proporciona uma compreensão significativa dos custos do seguimento ambulatorial pós-transplante de fígado por IHA e a participação de cada componente de custo no cenário brasileiro.

## INTRODUCTION

Brazil has a large public program of organ and tissue transplantations. According to the Brazilian transplant registry, 6,839 solid organ transplantations, among which 1,492 liver transplantations, were performed within the Brazilian National Health System (Sistema Único de Saúde, SUS) in 2011.^1^

The financial viability of high-cost procedures with small population coverage, such as organ transplantations, is an issue in low and middle-income countries that have limited resources and many competing priorities. Resource scarcity is a reality in all healthcare systems. Because of this scarcity, efficient allocation of resources is essential. The awareness of the importance of evaluating value for money in healthcare has increased over recent years.

Costs data on liver transplantations is scarce in Brazil, but the hospital costs of the transplantation procedure seem to be lower than in developed countries. In a study conducted in Fortaleza, state of Ceará, in northeastern Brazil, the hospital costs of the liver transplantation procedure ranged from US$ 11,384.30 to US$ 54,698.34, with a mean of US$ 20,605.01 in July 2008.^2^ However, the hospital costs of the procedure may have been underestimated in the study, which involved a retrospective analysis on medical records. In a prospective study, involving 24 patients who received transplants in the state of Paraná, southeastern Brazil, the hospital costs of the liver transplantation procedure ranged from US$ 21,582.90 (deceased donor) to US$ 22,986.60 (living donor), in January 2004.^3^ The study may also have underestimated the total hospital costs of the procedure since the costs of the healthcare workers and use of equipment were not included. The reimbursement paid by SUS for a liver transplantation, in December 2008, was R$ 57,089.41 (US$ 24,428.50), which included the hospitalization for the procedure, the surgery and the healthcare professionals involved.^4^

Besides hospitalization and procedure costs, outpatient care costs following transplantation should also be taken into consideration. After hospital discharge, the patient who received the transplant is followed up through outpatient care, with varying regularity according to clinical conditions and the institution’s protocol. Most patients receive immunosuppression throughout their lives. However, continued use of immunosuppression carries inevitable consequences: an increased risk of infections; metabolic complications such as hypertension, diabetes *mellitus* (DM), hyperlipidemia, obesity and gout; and cancers. Complications vary according to the time that has elapsed since transplantation: infections, perioperative causes and graft rejection account for most complications in the first year, whereas cardiovascular and metabolic diseases and malignancies are the leading cause of morbidity late after transplantation.^5^ The burden of healthcare utilization after liver transplantation may be substantial. Medical visits and routine laboratory tests are usually performed every month in the first semester, and every two or three months thereafter.

We conducted a cost descriptive study in order to estimate the direct medical costs of the outpatient follow-up after liver transplantation due to acute liver failure (ALF), from the first outpatient visit after transplantation to five years after transplantation, in a university hospital. The study was based on detailed retrospectively collected data on the outpatient visits, diagnostic tests, medications used and hospital admissions of 20 patients who underwent liver transplantation due to acute liver failure at the Digestive System Organ Transplantation Service of Hospital das Clínicas (HC), which is a tertiary hospital attached to the School of Medicine of the University of São Paulo (Faculdade de Medicina da Universidade de São Paulo, FMUSP), in São Paulo, Brazil.

## OBJECTIVE

Cost data relating to outpatient follow-up care after liver transplantation is scarce in Brazil, as well as in the international literature. To address this question, the objective of this study was to estimate the direct medical costs of the outpatient follow-up after liver transplantation, from the first outpatient visit after transplantation to five years after transplantation.

## METHODS

### Study population

The study population included adults who underwent liver transplantation due to acute liver failure (ALF), at the Digestive System Organ Transplantation Service of HC, between January 1, 2005, and December 31, 2009, who returned for at least one outpatient visit after hospital discharge. Thirty-eight patients were identified in a list provided by the service. Eighteen patients were excluded (15 died during the hospital stay for the liver transplantation procedure and three did not return to the outpatient clinic after discharge). The hospital data on all the 20 patients who returned to the outpatient clinic were reviewed.

### Outpatient follow-up protocol

During the study period, there was no strict protocol for immunosuppression. Generally speaking, immunosuppression was based on an association of tacrolimus and corticosteroids, with withdrawal of corticosteroids no later than six months after transplantation. The exceptions were patients with renal failure, who received triple immunosuppression (including mycophenolate mofetil), and individuals with autoimmune hepatitis, for whom triple immunosuppression was also used and corticosteroids were maintained. Medical visits and blood tests (hepatic panel, other biochemical tests and serum assaying of the immunosuppressive drugs) were scheduled weekly over the first months after transplantation, monthly until the end of the first year and then every three or four months if there were no complications.

### Data collection

Data were retrospectively retrieved from the patients’ medical records and the hospital accounting information system.

A structured form was used to collect the following demographic and clinical data from the medical records: age, sex, cause of ALF, comorbidities, date of liver transplantation, the first and last clinical outpatient visit, post-transplantation hospitalizations and length of stay, complications, immunosuppressive drugs and other medications used during outpatient care, diagnostic tests and medical visits (in the transplantation unit and other specialties), from the first outpatient medical visit to the last outpatient visit.

Data collection was conducted between December 2010 and January 2011.

### Economic evaluation

This economic analysis took the perspective of the healthcare provider and only included direct medical costs relevant to the healthcare service. Direct medical costs were calculated for each of the five years of follow-up.

Direct medical costs were estimated using “top-down” and “bottom-up” cost calculation methodology approaches. The top-down approach was used for the calculation of hospitalization costs; the bottom-down, for the other direct medical costs. Hospitalization costs include all costs relating to procedures, diagnostic test and medication that were incurred during hospitalization. In the top-down or gross-costing approach, all relevant cost components are identified at a highly aggregated level and valued per average per patient. The estimation of the resource utilization and costs is done based on information available on national administrative databases, such as the Diagnostic Related Groups (DRGs) in the United States or the Autorizações de Internações Hospitalares (AIH) paid by the Ministry of Health to hospitals in Brazil. In this study, the specific costs of each hospitalization were retrieved from the hospital accounting information system and corresponded to the reimbursement made by the Ministry of Health, through SUS (Sistema Único de Saúde), to the hospital. In the bottom-up or micro-costing approach, each component of resource use (for example, medications, diagnostic tests, medical visits) is identified and measured, and a unit cost is applied for each individual patient.

With regard to medications, immunosuppressive drugs, antibiotics and antiviral agents used for treatment or prophylaxis of frequent infections, and other drugs more frequently used were all included. Antihypertensive drugs were included in the analyses if the patient had not been hypertensive before the transplantation. Hypoglycemic drugs were not included if the patient had been using these drugs before the treatment. Less frequently used drugs, such as those used to treat specific infections, painkillers and antiemetics, were included in “other drugs”. Medication costs were estimated taking into account the dosages extracted from the medical records. The unit prices were calculated based on a drug-price guide from the hospital pharmacy.

The unit costs of visits to physicians and diagnostic tests were obtained from the public healthcare information system (SUS Management System for the Table of Procedures, Medications, Orthoses, Prostheses and Special Materials; Sistema de Gerenciamento da Tabela de Procedimentos, Medicamentos, Órteses e Próteses e Materiais Especiais do SUS, SIGTAP).^4^

Healthcare utilization was identified and costs were presented as the average cost per patient at the end of each year of the first five years post-transplantation. Patients’ information for each period was included provided that they had been followed up for at least 11 months of the respective year. In cases of death, the costs of treatment were incorporated independent of the length of follow-up in the respective year. Resources used and related costs were calculated per patient by multiplying the number of units used by the defined unit cost for each year of follow-up.

The costs presented are the sum of all the mean costs, per patient and year of follow-up. All costs are presented in 2008 reais (the Brazilian currency, US$ 1 = R$ 2.33, in December 2008) and were discounted at 5% per year.

## RESULTS

### Patient characteristics

The demographic characteristics, causes of ALF and preexisting conditions among the 20 adults with liver transplants who were included in this study are presented in Table 1. The main cause of ALF was drug-induced liver injury, which was observed in 9 cases (45%). The drugs relating to ALF in these patients were: methyldopa (4 cases), propylthiouracil (PTU) (2), isoniazid (2) and anesthetics (1). All the 20 patients included in the study underwent deceased-donor liver transplantation.

**Table 1.**
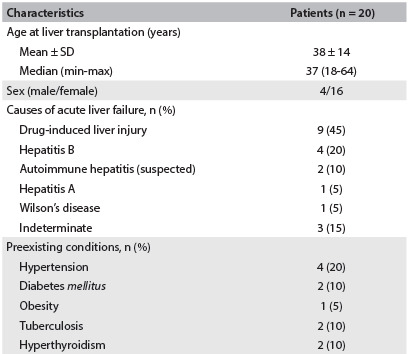
Characteristics of adults in outpatient care after liver transplantation due to acute liver failure at Hospital das Clínicas (HC), Faculdade de Medicina da Universidade de São Paulo (FMUSP), Brazil 2005-2009

SD = standard deviation.

The length of outpatient follow-up after transplantation varied according to the date of the liver transplantation. Two patients were lost (after one and four years of outpatient follow-up) and one patient died (in the 8^th^ month after transplantation). The other 17 patients were still being followed up as outpatients at the time of data collection. Thus, for the first year after transplantation, healthcare resource utilization and cost estimates were based on data from all 20 patients. For the subsequent years, the estimates were based on information from 14 patients (second year), 12 patients (third year), 7 patients (fourth year) and 3 patients (fifth year).

### Complications during follow-up after transplantation

Eight episodes of infection were diagnosed during follow-up: cytomegalovirus disease (2 episodes), pulmonary aspergillosis (1), central nervous system tuberculosis (1), orolabial herpes (1), parvovirus B19 infection (1), pneumonia (1) and acute appendicitis (1).

Three patients presented an episode of transplant rejection during the first year after transplantation.

Other complications included anemia (5 episodes), incisional hernia (4), biliary stenosis (3) and chronic renal failure (2).

### Healthcare resource utilization

#### Medications

The immunosuppressive drugs used by all the patients during the entire period were the main cost driver (Table 2), followed by the drugs used for treatment or prophylaxis of infections: antiviral agents (ganciclovir and valganciclovir) for treatment or prophylaxis of cytomegalovirus; hepatitis B prophylaxis; and sulfamethoxazole-trimethoprim, mainly used in the first two years of follow-up. There was also a decrease in the frequency and costs of drugs over time. The decrease in costs of immunosuppressive drugs was associated with discontinuation of corticosteroids and reduced doses of tacrolimus and cyclosporine.

**Table 2.**
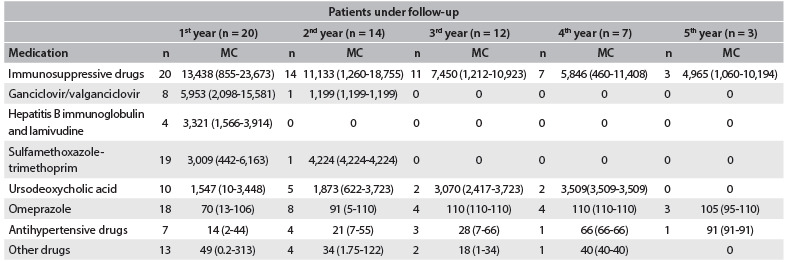
Number of patients under treatment and mean costs of medications according to the year of follow-up (in Brazilian reais)

n = Number of patients in treatment; MC = Mean costs (min-max).

#### Diagnostic tests

The following laboratory tests were performed for all patients every year: hepatic panel (alanine aminotransferase, ALT; aspartate aminotransferase, AST; gamma glutamyl transpeptidase, γGT; alkaline phosphatase, ALP; bilirubins and coagulogram), other biochemical tests (serum urea, creatinine, sodium, calcium, potassium and glucose), blood cell count and serum assays on immunosuppressive drugs (cyclosporine and tacrolimus). The number of times that these tests were done also decreased during the follow-up and ranged from 16to 53 (mean: 27.6) in the first year after transplantation; 5 to 37 (mean: 14.4) in the second year; 5 to 18 (mean, 8.9) in the third year; 4 to 9 (mean: 6.9) in the fourth year; and 3 to 6 (mean: 4.6) in the fifth year.

The frequencies of other diagnostic tests, such as radiographs, abdominal ultrasound, echocardiography, computed tomography (CT), cholangiopancreatography resonance, bone densitometry, scintigraphy, endoscopy and biopsy, which were not routinely performed for all patients, also decreased over time (Table 3).

**Table 3.**
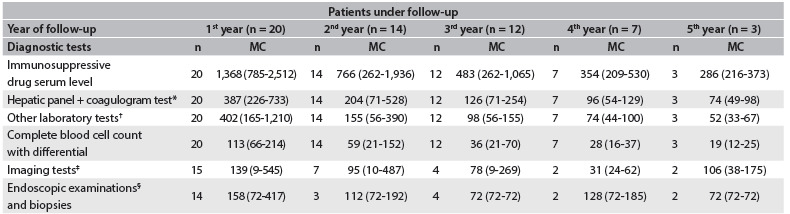
Numbers of patients undergoing diagnostic tests and test costs according to the year of follow-up (in Brazilian reais)

n = Number of patients in treatment; MC = Mean costs (min-max). *Hepatic panel: alanine aminotransferase (ALT), aspartate aminotransferase (AST), gamma glutamyl transpeptidase (γGT), alkaline phosphatase (ALP), bilirubins (total, direct and indirect). Coagulogram test = (prothrombin time + INR); ^†^Other laboratory tests: serum urea, creatinine, sodium calcium, potassium, glucose and hepatitis B; ^‡^Imaging: radiographs, abdominal ultrasound, echocardiography, computed tomography (CT), cholangiopancreatography resonance, bone densitometry, scintigraphy; ^§^Endoscopic examinations: endoscopy, colonoscopy, enteroscopy, bronchoscopy.

Serum assaying of immunosuppressive drugs was the main driver of diagnostic test costs. Although hepatic panels and other biochemical tests were not individually expensive, these also led to substantial costs due to the high frequency with which they were repeated.

#### Medical visits

The number of medical visits decreased over time, ranging from 13 to 43 (mean: 23) in the first year of follow-up after transplantation; from 4 to 35 (mean: 13.2) in the second year; from 3 to 16 (mean: 8) in the third year; from 5 to 10 (mean: 7.8) in the fourth year; and from 3 to 10 (mean: 6.3) in the fifth year.

#### Hospitalizations

In the first year of outpatient follow-up, 14 post-transplant patients (70%) were hospitalized, thus totaling 30 hospital admissions (1-6/patient). The length of hospitalizations ranged from 1 to 29 (mean: 13.4) days. There were decreases in the numbers of hospital admissions in the subsequent years. In the second year of follow-up, six patients had 10 hospital admissions (1-4/patient) and the duration of hospitalizations ranged from 1 to 16 days (mean: 6). In the third year after transplantation, three patients had five hospital admissions (1-3/patient); the length of hospitalizations ranged from 1 to 4 days (mean: 2). In the fourth year, there was one hospitalization (2 days in length). No hospitalizations occurred in the fifth year of follow-up after transplantation. The main cause of hospital admissions was “abnormal liver enzymes”, which was responsible for 11 events during the 5-year follow-up; followed by fever (5 hospital admissions), biliary drainage (4) and abdominal pain (4). The other causes of hospital admissions were diarrhea and vomiting (3), diabetes *mellitus* (3) and incisional hernia repair (3).

### Direct medical costs

The mean total costs and minimum and maximum values for the five-year period after transplantation are presented in Table 4.

**Table 4.**
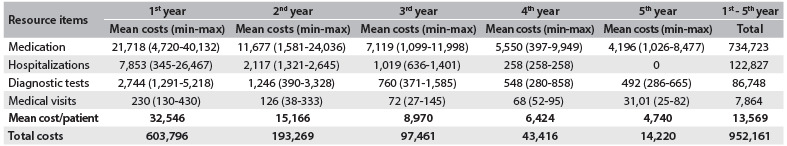
Mean and total direct medical costs incurred per patient according to year of follow-up*, 2005-2009

*The estimates were based on data from 20 patients (1^st^ year), 14 patients (2^nd^ year), 12 patients (3^rd^ year), 7 patients (4^th^ year) and 3 patients (5^th^ year).

**Figure 1.**
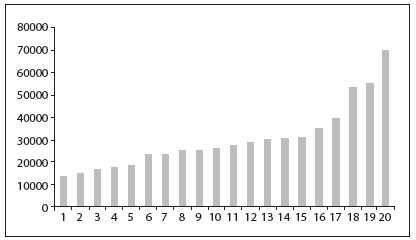
Total direct medical costs in the first year after transplantation, for all 20 patients.

**Figure 2.**
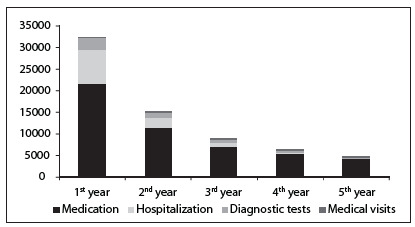
Summary breakdown of mean direct medical costs incurred per person per resource use per year of outpatient follow-up after liver transplantation.

The total cost of all 20 patients over the five-year period follow-up after liver transplantation at our hospital was R$ 952,161 (US$ 408,653). The mean cost per patient/year was R$ 13,569 (US$ 5,824). In the first year after transplantation, the mean total cost of follow-up was R$ 32,546 (US$ 13,968) and presented a large variation, ranging from R$ 6,486 (US$ 2,784) to R$ 72,247 (US$ 31,007) per patient. There was a left-skewed gamma distribution in the mean direct costs (Figure 1). In the second year after transplantation, the mean total costs were about half of the amount of the first-year costs (R$ 15,165 or US$ 6,509) and ranged from R$ 3,330 (US$ 1,429) to R$ 30,342 (US$ 13,022) per patient. The higher costs in the first year of follow-up can be attributed to higher spending on hospitalization and medication. Hospitalization contributed 18% of all the direct medical costs. In the second year after transplantation, the contribution of hospitalization fell to approximately 6%. Medication accounted for approximately 85% of the total costs throughout the follow-up period. Figure 2 shows the major cost drivers during the post-transplantation follow-up. Medication was the largest contributor to the costs, followed by hospitalization. In the fourth year, diagnostic tests costs surpassed the hospitalization costs.

## DISCUSSION

The total direct cost over the five-year follow-up period after liver transplantation for these 20 patients in a tertiary-level university hospital in São Paulo was R$ 952,161 (US$ 408,653). The first year of follow-up was the most expensive and medication was the main driver of the total costs, accounting for 85% of the total costs over the five-year follow-up period, and 71.9% of the first-year total costs. As expected, the costs decreased over the years due to reductions in the numbers of immunosuppressive drugs and dosages used, as well as declining hospitalization. In the third and fourth years, ursodeoxycholic acid, with low unit cost but continuous use, became the second largest cost among the medications.

In Brazil, only data on the hospital costs of the transplantation procedure have been published.^2^^,^^3^ The costs involved in hospitalization are relatively simple to estimate, but the subsequent phases can be extremely variable in terms of healthcare utilization, and hence costs, because of differences in patient severity and surgical outcomes. To our knowledge, our study was the first to evaluate the costs of outpatient follow-up after liver transplantation in Brazil.

Direct comparison of costs between different studies is difficult due to differences in the healthcare systems of different countries and the time periods analyzed.^6^ Nonetheless, our findings are similar to what has been reported in some international studies. In a study in Taiwan that evaluated the total costs of outpatient care over the first year after kidney, heart and lung transplantation, using a countrywide health claims database, drugs were the main component of outpatient costs and were responsible for around 80-90% of the total healthcare costs. In relation to liver transplantation, medication was responsible for 85.75% of the total costs over the first year of follow-up, whereas the estimate in our study was 71.9%.^7^ In another study that prospectively evaluated the costs starting from inclusion in the liver transplant waiting list to the end of the first year after transplantation, in a French university hospital, drug expenses accounted for 70% of outpatient care costs.^8^

The majority of the studies that have evaluated outpatient costs took into consideration the costs of the first year after the surgical procedure. In our study, we evaluated the costs over a follow-up period of five years after liver transplantation. The costs were calculated using the bottom-up approach (except for hospitalizations, for which the hospital charges were used), which allowed us to identify all the cost elements individually.

Our study only included patients with ALF. Liver transplant due to ALF has high early mortality and graft loss, particularly within the first year after transplantation.^9^ After this critical period, these patients’ long-term prognosis is quite good. In a study comparing the costs of transplantation procedures and the first-year follow-up, patients with ALF had 42% higher costs than patients with chronic disease. This difference resulted mainly from hospitalization, intensive care and support system costs (molecular adsorbent recirculating system). However, over the subsequent two to five years, the costs were lower among ALF patients. Immunosuppressive drug costs were significantly higher among chronic patients.^10^

The estimates constructed in our study are essential for conducting a Brazilian model-based cost-effectiveness analysis. Data from cost estimates are considered to have low transferability. The economic evaluation guidelines are very strict and do not allow these data to be transferred under any circumstances. Cost unit estimates must be specific to the context under evaluation, due to differences in absolute and relative prices between countries.^11^ A Brazilian model will require the pattern of care practiced in local health services in order to determine the resources (medical consultations, hospitalizations, diagnostic tests, medications, etc.), and the amounts used in outpatient, inpatient and transplant cases and in post-transplantation follow-up.^12^

Our study has several limitations. Although we analyzed all the patients who received transplants due to ALF and who were being followed up at our center during the period studied, the sample size was small. This is a limitation because of the diversity of causes of liver transplantation and the possible clinical evolution, complications and sequelae after transplantation. There were smaller numbers of patients in the last two years of follow-up: seven patients in the fourth year and three patients in the fifth year.

Hospitalizations were important cost drivers in economic evaluations, but because of the lack of detailed (patient level) data, the top-down approach was the only feasible option. The top-down approach has been used in several countries, including Australia, Belgium, Sweden, United Kingdom and the United States, to calculate hospital treatment costs. Moreover, the top-down approach is cheaper and faster than the bottom-up approach.^13^ The bottom-up methodology enables detection of costs differences between patients of each single component of resource use. This methodology is time consuming, mainly when hospital information systems are absent or inadequate, and it has not been widely used because of its low feasibility. The bottom-up method can be highly accurate but expensive to use. On the other hand, the top-down method is more feasible, but its disadvantage is that it fails to trace costs of specific patients. Therefore, cost differences between patients cannot be revealed. The bottom-up and top-down methods are not mutually exclusive. In fact, it is often appropriate to use both methodologies in the same study.

The SUS reimbursement fees used in this study might be a useful approximation, which may be more relevant from a national perspective than costs calculated within that particular hospital.

Another limitation of the study is that it evaluated just one center, a teaching hospital in São Paulo, which is the biggest city in Brazil. Teaching hospitals that focus on research and education are considered costly in relation to other hospitals. This center in São Paulo is not representative of all Brazilian transplantation centers. It is difficult to generalize the costs of follow-up after transplantation from this center to other states in Brazil, which is a heterogeneous country with differing realities and social conditions. In other centers, the healthcare resource utilization may be less intensive, with fewer diagnostic tests and less expensive drugs in routine practice care.

## CONCLUSIONS

We studied only the direct medical costs of the follow-up. Direct non-medical and indirect costs, which are an important part of the costs after transplantation, were not addressed in the present study, because of the complexity of the methodology involved. Despite these caveats, this analysis provides significant insight into the costs of outpatient follow-up after liver transplantation and the participation of each cost component in the Brazilian setting. Further studies need to be conducted in multiple transplant centers, with bigger samples, including patients who received transplants due to chronic liver disease, and direct non-medical and indirect costs. Economic evaluation methods in healthcare can be an important tool for assessing the costs of health technologies and helping policy makers inform efficient funding allocations. Knowing these costs is the first step towards establishing a specified threshold, identifying cost outliers and reducing costs, in a country where choices and priorities need to be set.
